# Neonatal Acute Liver Failure due to Citrin Deficiency (NALFCD)

**DOI:** 10.1002/jmd2.70110

**Published:** 2026-07-22

**Authors:** Hoi‐Yin Chan, Rosanna Wong, Cheuk‐Wing Fung, Kiran Moti Belaramani, Chloe Miu Mak, Matthew Yeung, Toby Chan, Carmen Yeung, Yvonne Kwok, Anne Mei‐Kwun Kwok

**Affiliations:** ^1^ Department of Pediatrics and Adolescent Medicine Hong Kong Children's Hospital Hong Kong China; ^2^ Faculty of Medical Sciences Radboud University Nijmegen the Netherlands; ^3^ Newborn Screening Laboratory, Department of Pathology Hong Kong Children's Hospital Hong Kong China; ^4^ Dietetics Team, Integrated Rehabilitation Centre Hong Kong Children's Hospital Hong Kong China

**Keywords:** citrin deficiency, citrullinemia type I, neonatal acute liver failure, newborn screening

## Abstract

Both citrin deficiency (CD) and citrullinemia type I (CTLN1) may be detected by elevated citrulline through newborn screening (NBS) or present as acute liver failure in later infancy, but they differ significantly in management. This may pose therapeutic challenges in the early period after presentation while awaiting diagnostic confirmation. We report a Chinese girl (birth weight 1.98 kg at 37 weeks) who was recalled on day 5 for elevated citrulline (47 μmol/L; cutoff < 25) and citrulline/arginine ratio of 7.34 at NBS on day 2. Citrulline rose to 264 μmol/L upon retesting on day 5. Initial investigations revealed INR of 3.4 and raised alkaline phosphatase, while ammonia, conjugated bilirubin, glucose, albumin, gamma‐glutamyl transferase, and transaminases were normal. Suspected CTLN1 led to halting protein intake and starting high glucose infusion. Within 17 h, INR increased to 6.5 and albumin dropped. Worsening hepatic function following high‐glucose intake and her small for gestation age status suggested CD. She improved rapidly after switching to lactose‐free MCT‐enriched formula. Genetic analysis revealed compound heterozygous known pathogenic mutations in the *SLC25A13* gene, confirming the diagnosis of CD. This is the first report of CD presenting with neonatal acute liver failure without cholestasis. It highlights the importance of prompt differentiation between CD and CTLN1 in NBS recalls for safe and effective interim treatment.

## Take‐Home Message

Citrin deficiency (CD) can cause neonatal acute liver failure (NALF) without cholestasis, highlighting the need for:

- Early consideration of CD in NALF, as standard management strategies for NALF are harmful to neonates with CD.
- Close monitoring and prompt redirection of interim treatment for neonates with elevated citrulline at newborn screening awaiting diagnostic confirmation could rapidly reverse liver failure.

## 1 Introduction

Citrin deficiency (CD) or citrullinemia type II is an autosomal recessive metabolic disorder due to a loss of function in the *SLC25A13* gene, which encodes for the protein citrin. It affects multiple metabolic processes, including glycolysis, gluconeogenesis, galactose metabolism, urea cycle and fatty acid synthesis, and presents with a spectrum of clinical manifestations at different ages, from neonatal intrahepatic cholestasis associated with citrin deficiency (NICCD), to failure to thrive and dyslipidemia due to citrin deficiency (FTTDCD) in childhood, and adolescent/adult‐onset citrullinemia type II (AACD) with acute hyperammonemia and liver failure. Rarely, infants with NICCD might have progressive liver failure requiring transplantation [1].

Previously considered as a predominantly East Asian disease, CD is now recognized as a pan‐ethnic disorder. Some regions have included CD in the newborn screening (NBS) programs [2], which enable early recognition and treatment of this condition. Detection of CD through NBS can be challenging using elevated citrulline levels as marker: (1) It is not specific to citrin deficiency—it is also used to screen distal urea cycle disorders (d‐UCD) and some newborns with CD have high citrulline levels overlapping with those having d‐UCD [3]. (2) Its sensitivity is also low—affected newborns could have citrulline levels overlapping with the normal population [1].

The current treatment for CD involves a diet low in carbohydrates, high in protein, and supplemented with medium chain triglycerides (MCT) [1]. Treatment for d‐UCD, including protein restriction and high carbohydrate provision [4] is contraindicated for CD. This poses management challenges for newborns who are screened to have high citrulline levels and undergo diagnostic workup.

Herein, we report a newborn girl with CD identified through NBS. At the time of recall, the patient had already developed significant coagulopathy. We detail the initial presentation and management and the subsequent diagnosis and treatment. This case highlights the management complexities during the initial period following NBS for high citrulline before diagnostic confirmation, and alerts clinicians to include CD as a differential diagnosis of neonatal acute liver failure (NALF).

## 2 Case Presentation

**FIGURE 1:**
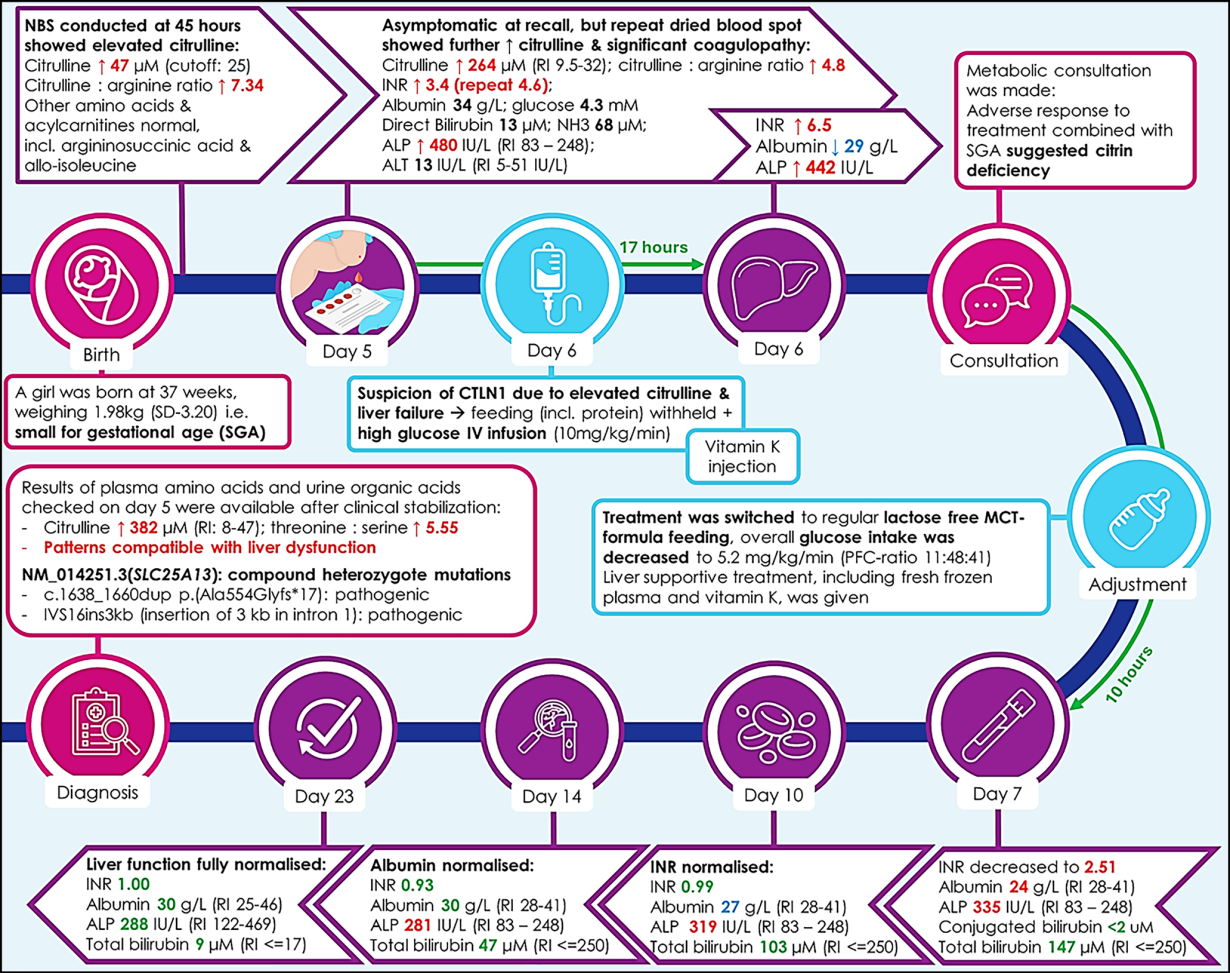
Time course before diagnostic confirmation.

A girl was born at 37 weeks of gestation via normal spontaneous delivery with a birth weight of 1.98 kg and standard deviation (SD) of −3.20. NBS was conducted at 45 h of life. She was recalled on day 5 of life, as NBS revealed elevated citrulline (47 μmol/L; cutoff: 25 μmol/L) with a citrulline to arginine ratio of 7.34. Other amino acids and acylcarnitines were unremarkable. No other abnormality was detected including argininosuccinic acid and allo‐isoleucine (Figure 1).

She was asymptomatic at the time of recall. Repeat of dried blood spot on day 5 of life showed further elevation of citrulline to 264 μmol/L, with citrulline to arginine ratio of 4.8. Liver assessment revealed significant coagulopathy with international normalized ratio (INR) of 3.4, prothrombin time (PT) of 36.6 s (reference interval or RI: 10.5–13.0 s) and activated partial thromboplastin time (APTT) of 72.7 s (RI: 23.0–34.7 s). Alkaline phosphatase was slightly elevated (480 IU/L; RI: 90–273 IU/L). Other liver biochemistries were unremarkable including blood glucose, albumin, ammonia, bilirubin, gamma‐glutamyl transferase (GGT) and alanine transaminase. She was suspected to have citrullinemia type 1 (CTLN1) and given high glucose infusion with a glucose infiltration rate (GIR) of 10 mg/kg/min. Protein was withheld. Within 17 h, INR increased up to 6.5 despite vitamin K1 injection and albumin dropped to 29 g/L. Other liver parameters remained static.

Metabolic specialist consultation was made. The acute worsening of liver synthetic function after empirical glucose treatment, together with the background of small for gestation age (SGA) which is common among babies with CD [1], suggested CD as the more likely diagnosis than d‐UCD. She was switched from intravenous high glucose infusion to regular feeding with lactose‐free MCT‐based formula with an overall glucose intake of 5.2 mg/kg/min. INR decreased to 2.51 within 10 h, and normalized in 3 days. Albumin and alkaline phosphatase also normalized in 7 and 10 days, respectively.

Results of plasma amino acids and urine organic acids performed on day 5 of life were available after stabilization. They revealed patterns compatible with that of CD and liver injury—elevated plasma citrulline (382 μmol/L; RI: 8–47 μmol/L), threonine (588 μmol/L; RI: 40–204 μmol/L, with threonine to serine ratio of 5.55), methionine (70 μmol/L; RI: 13–43 μmol/L), ornithine (193 μmol/L; RI: 20–136 μmol/L), arginine (128 μmol/L; RI: 12–112 μmol/L), phenylalanine (254 μmol/L; RI: 26–98 μmol/L) and tyrosine (380 μmol/L; RI: 19–119 μmol/L); and moderate hyperexcretion of tyrosine and phenylalanine metabolites in urine. The other plasma amino acids and urine metabolites were within normal range. Genetic analysis subsequently confirmed compound heterogenous known pathogenic mutations in the *SLC25A13* gene—c.1638_1660dup (p.Ala554Glyfs*17) and IVS16ins3kb (insertion of 3 kb in intron 1).

## 3 Discussion

To our best knowledge, this is the first reported case of NALF due to CD. Our case shows CD could present with acute liver failure (ALF) as early as the first few days of life. NBS had enabled early diagnosis of CD and prevented potential catastrophic complications and need of liver transplantation in this newborn. This case also highlights the challenges in clinical management of babies presenting with elevated citrulline in NBS awaiting diagnostic confirmation.

Across the different life stages of patients with CD, acute liver failure is primarily reported in adulthood or late adolescence as a manifestation of AACD. It is not reported in the neonatal period and is rarely observed as the presenting manifestation in other age groups. One infant was reported to present with ALF at 8 months of age, with severe normal‐GGT cholestasis and coagulopathy, moderate hyperammonemia, and only mildly raised transaminases [5]. Rare NICCD cases with progressive liver failure after diagnosis share a similar pattern of hepatic dysfunction [1, 6].

Although NBS facilitates early recognition of CD, elevated citrulline—the primary biomarker utilized—is non‐specific. It also serves as the biomarker to screen d‐UCD, and a variety of other inherited metabolic disorders can also cause secondary elevation of citrulline in NBS (Table 1, Figure S1). While some of these conditions have easily distinguishable features for example, elevated argininosuccinate in dried blood spot and urine for argininosuccinic aciduria (ASA), elevated urinary lysine for lysinuric protein intolerance (LPI), or lactic acidosis and neurological signs for pyruvate carboxylase deficiency (PCD), differentiating other disorders solely based on citrulline levels remains challenging. In CD, NBS citrulline levels range from normal to greater than 500 μmol/L. Some affected babies show only slight elevation on the initial screen but a dramatic increase upon re‐screening [7]. In CTLN1, the classical early‐onset form is characterized by profoundly elevated citrulline level and early‐onset symptomatic hyperammonaemia within the first weeks of life, while the non‐classical late‐onset form and heterozygous state are typically associated with mild citrulline elevation of 40–100 μmol/L [8]. Notably, majority of neonates with CTLN1 detected in NBS belong to the attenuated type, and usually have normal ammonia levels at presentation [9].

**TABLE 1:** Differential diagnosis of elevated citrulline in newborn screening.

	Citrin deficiency (CD)	Citrullinemia type I (CTLN1)	Arginino‐succinic aciduria (ASA)	Lysinuric protein intolerance (LPI)	Pyruvate carboxylase deficiency (PCD)
Citrulline levels	Normal to elevated	Mildly to severely elevated	Mildly elevated	Mildly elevated (secondary finding)	Elevated (secondary finding)
Bio‐chemistry	– Elevated ammonia – Elevated plasma methionine, arginine, ornithine, phenylalanine, threonine & tyrosine – Galactosuria	– Elevated ammonia – Low‐to‐normal plasma arginine & ornithine – Absent argininosuccinate (ASA) in blood & urine	– Elevated ammonia – Elevated ASA in blood & urine	– Elevated ammonia – Elevated urine cationic amino acids, especially lysine	– Elevated ammonia – Elevated plasma alanine, lysine & proline – Elevated lactate
Age of onset	Neonatal to adulthood	Neonatal to adulthood	Neonatal to adulthood	Infancy to childhood	Neonatal to childhood
Clinical features	– Small for gestational age, failure to thrive, chubby face – Neonatal cholestasis, liver dysfunction (mainly synthetic in infancy), fatty liver – Acute liver failure reported	– Normal at birth – Lethargy, poor feeding, vomiting, neurocognitive deficiencies – Liver dysfunction, hepatomegaly – Acute liver failure reported	– Normal at birth – Lethargy, poor feeding, vomiting, neurocognitive deficiencies – Liver dysfunction, hepatomegaly – Hypertension, coarse brittle hair	– Normal at birth; failure to thrive – Poor feeding, vomiting, diarrhea, hypotonia, hepatomegaly	– Hypothermia, respiratory distress or even failure, vomiting, neurological symptoms, hepatomegaly
Gene	*SLC25A13*	*ASS1*	*ASL*	*SLC7A7*	*PC*

Acute liver failure has been reported as the presenting feature of CTLN1 both in infancy and in adulthood particularly during pregnancy [4]. Notably, elevations in transaminases and INR may not always be accompanied by hyperammonemia. In ASA, chronic liver dysfunction is common and characterized by persistent elevation of plasma alanine transaminase activity and hepatomegaly [10], however, isolated ALF has not yet been reported. Liver involvement seldom occurs in isolation and is not the most predominant feature in LPI and PCD.

When a baby is screened to have elevated citrulline, prompt diagnosis and initiation of appropriate nutritional management are essential to prevent exacerbation of underlying metabolic disturbances and serious complications including ALF, as well as irreversible liver and neurocognitive damages. Management strategies for CD are quite opposite to that for CTLN1/ASA (Table 2). In CD, carbohydrate is not well tolerated as a result of impaired sugar metabolism. Cell and animal studies show impaired glycolysis and ATP generation due to disrupted NADH/NAD^+^ balance in both cytosol and mitochondria [1]. Patients rely on alternative energy sources including protein and medium‐chain triglycerides. On the other hand, the key management for CTLN1/ASA is to have dietary protein restriction to minimize the nitrogen load with l‐arginine supplementation. High glucose provision is instrumental to suppress catabolism especially in young infants having high risk of acute metabolic decompensation. The overlapping features shared by both CD and d‐UCD especially CTLN1 could make the choice of interim nutritional management very challenging while teasing out these two differential diagnoses.

**TABLE 2:** Nutritional management for babies with CD, CTLN1/ASA, and neonatal acute liver failure (NALF) with unknown etiology.

Macronutrients	CD	CTLN1/ASA	NALF
Carbohydrate	Restricted	High	High
Protein	High	Restricted arginine supplementation	Adequate ± temporary restriction
Fat	High MCT supplementation	Normal	Normal
Administration	Avoid fasting		

Recent development of second‐tier genetic screening for CD mainly focuses on those who are detected to have high‐normal or borderline citrulline levels in NBS [7, 11]. It reduces the false negative rate of NBS for affected babies with high‐normal citrulline levels, and the false positive rate by enabling early differentiation of CD from ASS heterozygosity. Current second‐tier genetic screening strategies do not target infants with clearly high citrulline levels—instead of waiting for genetic screening results, these infants are recalled immediately for urgent empirical treatment and diagnostic evaluation [8, 11]. Further studies are needed to assess the operational feasibility of a concurrent workflow upon detecting high citrulline levels—initiating rapid genetic screening in parallel with emergency call‐back, to minimize empirical treatment duration.

While both CD and CTLN1 can be the differential diagnoses of ALF, especially in the context of elevated citrulline, it is important to keep a very close monitoring of patient response to empirical management before the diagnostic confirmation can be made. In case there is worsening of liver function, especially the synthetic aspects, after standard high glucose provision and protein restriction for NALF or suspected CTLN1, clinicians should be alert to the possibility of CD and consider a prompt re‐direction of treatment strategies to carbohydrate restriction, high protein diet, with MCT supplementation. It could reverse the liver damage and prevent the need for liver transplantation, as demonstrated by the quick improvement in our patient.

## 4 Conclusions

CD can cause NALF even in the absence of NICCD. It is important to closely monitor and timely adjust the interim treatment when there is no clinical improvement for babies with elevated citrulline at NBS awaiting diagnostic confirmation.

## Author Contributions

**Anne Mei‐Kwun Kwok:** conceptualization, methodology, project administration, supervision. **Hoi‐Yin Chan:** data curation, writing – original draft, visualization. **Hoi‐Yin Chan**, **Rosanna Wong**, **Cheuk‐Wing Fung**, **Kiran Moti Belaramani**, **Carmen Yeung**, **Yvonne Kwok:** data acquisition, validation. **Chloe Miu Mak**, **Matthew Yeung**, **Toby Chan:** investigation, data interpretation. All authors: writing – review and editing.

## Funding

The authors have nothing to report.

## Ethics Statement

Approval was obtained from the local human research ethics committee.

## Consent

Written informed consent was obtained from the parent of the reported patient.

## Conflicts of Interest

The authors declare no conflicts of interest.

## Supporting information


**Figure S1:** High citrulline in newborn screening: differential diagnosis and management.

## Data Availability

The data that support the findings of this study are available on request from the corresponding author. The data are not publicly available due to privacy or ethical restrictions.
