# Supplementary figures and images for: Neonatal Acute Liver Failure due to Citrin Deficiency (NALFCD)
Source: JIMD Rep. 2026 Jul 22;67(4):e70110. doi: 10.1002/jmd2.70110 (PMC13391237; doi:10.1002/jmd2.70110)

**
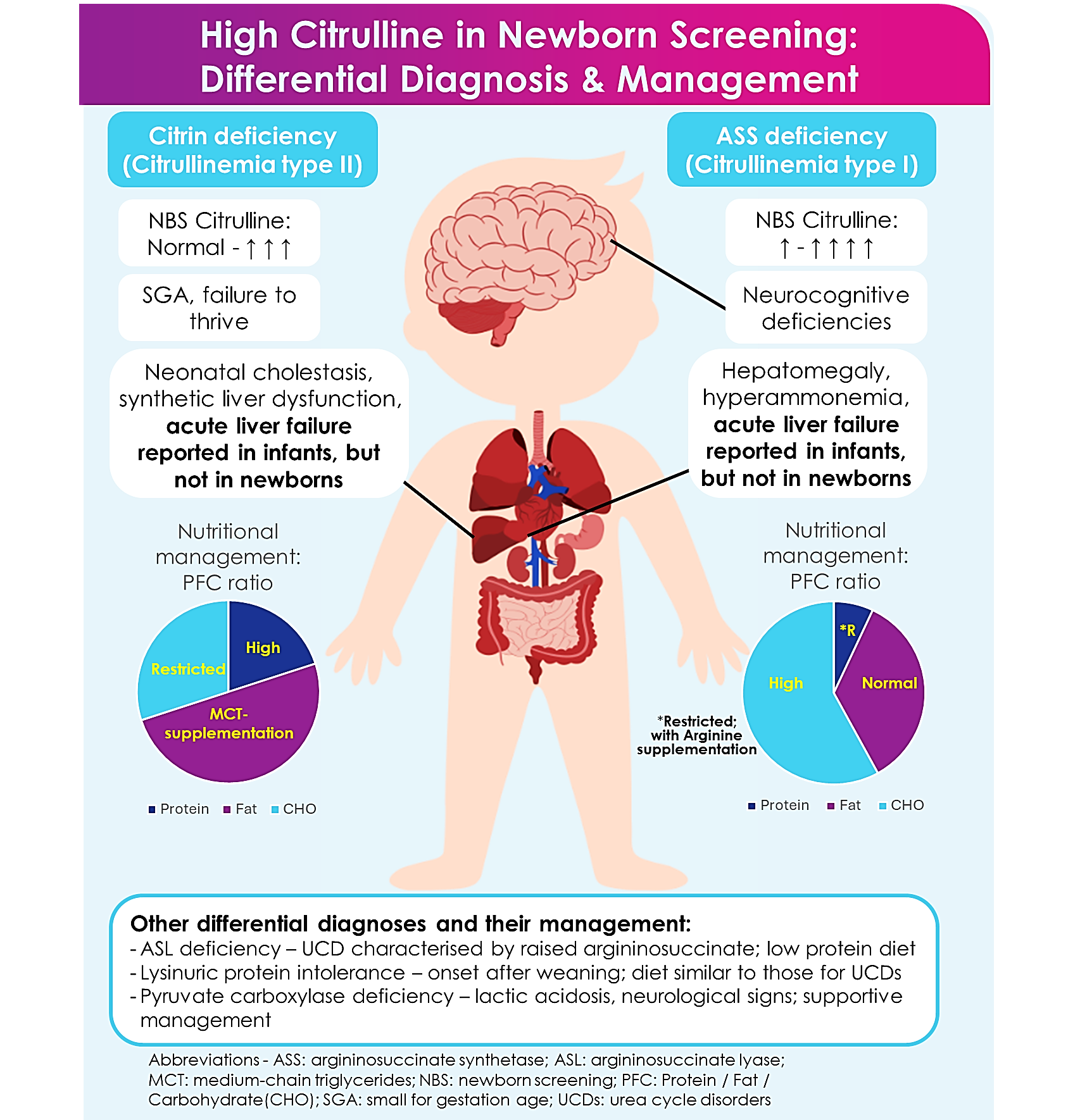
Figure S1: High citrulline in newborn screening: differential diagnosis and management**

Supplement: Supplementary file 1 — Figure S1: High citrulline in newborn screening: differential diagnosis and management. [file JMD2-67-e70110-s001.docx]
